# 

**Published:** 1858-10

**Authors:** 


*Buffalo Medical College.*—The regular lecture term in this institution, will commence on the first Wednesday in November, (3d.) The college will be opened from the first Wednesday in October to those who wish to pursue practical anatomy in advance of the lecture session. From this date, also, the attendance at the hospital by the officers of the college commences, and clinical instruction is given.

So far as an opinion can be formed in advance, the class for the coming session will be larger than last year. Ample provision has been made for those who, from motives of economy, are constrained to go to institutions where instruction is gratuitous or the fees merely nominal. While the faculty of the college do not hold to the doctrine of free teaching, they are willing to receive students who, from circumstances beyond their control, feel obliged to resort to schools which hold out this inducement. They who would elect the Buffalo school on equal pecuniary terms, will find it to their advantage to address the Dean on this subject.

The opportunities for clinical instruction in this school are so well known, that it is unnecessary to present them to our readers.

*Medical and Surgical Reporter.*—We see by an announcement in the last number ef this Journal, that it will be hereafter issued as a weekly. We congratulate the editors upon their good judgment in this regard. There are now quarterly, bimonthly and monthly Journals in Philadelphia, and there seems to be a much more favorable opening for a weekly than at any other point. We wish it all success in its new form.

---

*To our Subscribers, Paying and Delinquent.*—We will commence our dun to the delinquents by expressing our thanks to those subscribers who appreciate that a Journal of sixty-four pages cannot be issued without cost, and have so kindly sent us the amount of their subscription; but some are not yet aroused to this fact; and to such we must make an appeal of some kind. We are now asking merely for enough money to pay expenses, but if every delinquent would take this to himself, and mail immediately $2.50, we should have enough and more. The small amount of our subscription, can certainly be sent *immediately by every subscriber;* and if we can touch your conscience kind reader, when we call to your mind our *gratuitous* labors in your behalf, made the more weighty by anxiety in regard to meeting the actual cost of issue from the subscription list, send us our money before your sense of justice becomes more obtuse. One and all, owing for back subscriptions or not, send us the price of vol. XIV.

